# Dual-Specificity Phosphatase CDC25B Was Inhibited by Natural Product HB-21 Through Covalently Binding to the Active Site

**DOI:** 10.3389/fchem.2018.00531

**Published:** 2018-11-13

**Authors:** Shoude Zhang, Qiangqiang Jia, Qiang Gao, Xueru Fan, Yuxin Weng, Zhanhai Su

**Affiliations:** ^1^State Key Laboratory of Plateau Ecology and Agriculture, Qinghai University, Xining, China; ^2^Department of Pharmacy, Medical College of Qinghai University, Xining, China; ^3^School of Pharmacy, East China University of Science and Technology, Shanghai, China

**Keywords:** Cdc25B inhibitor, sesquiterpene lactone, anticancer, cell cycle progression, covalent binding to protein

## Abstract

Cysteine 473, within the active site of the enzyme, Cdc25B, is catalytically essential for substrate activation. The most well-reported inhibitors of Cdc25 phosphatases, especially quinone-type inhibitors, function by inducing irreversible oxidation at this active site of cysteine. Here, we identified a natural product, HB-21, having a sesquiterpene lactone skeleton that could irreversibly bind to cys473 through the formation of a covalent bond. This compound inhibited recombinant human Cdc25B phosphatase with an IC_50_ value of 24.25 μM. Molecular modeling predicted that HB-21 not only covalently binds to cys473 of Cdc25B but also forms six hydrogen bonds with residues at the active site. Moreover, HB-21 can dephosphorylate cyclin-dependent kinase (CDK1), the natural substrate of Cdc25b, and inhibit cell cycle progression. In summary, HB-21 is a new type of Cdc25B inhibitor with a novel molecular mechanism.

## Introduction

Dual-specificity protein phosphatases (DSP) such as Cdc25s (Cdc25A, Cdc25B, and Cdc25C) play an essential role in cell cycle progression by controlling the phosphorylation state of their natural substrates, cyclin-dependent kinases (CDKs) (Kristjánsdóttir and Rudolph, 2004). Overexpression of Cdc25s and overactivation of CDKs are involved in cancer-associated cell-cycle aberrations (Kristjánsdóttir and Rudolph, 2004). Therefore, Cdc25s have been demonstrated as promising anticancer targets (Boutros et al., 2006, 2007; Xing et al., 2008). Cysteine 473, within the active site of the enzyme Cdc25B, is catalytically essential for activating its natural substrates. Most potent small-molecule inhibitors of the Cdc25 phosphatases are quinone-derived compounds. It has been reported that inhibition of Cdc25B activity can occur by the oxidation of the cys473 through the production of reactive oxygen species (Brisson et al., 2007; Lavecchia et al., 2010, 2012).

**Figure 1:**
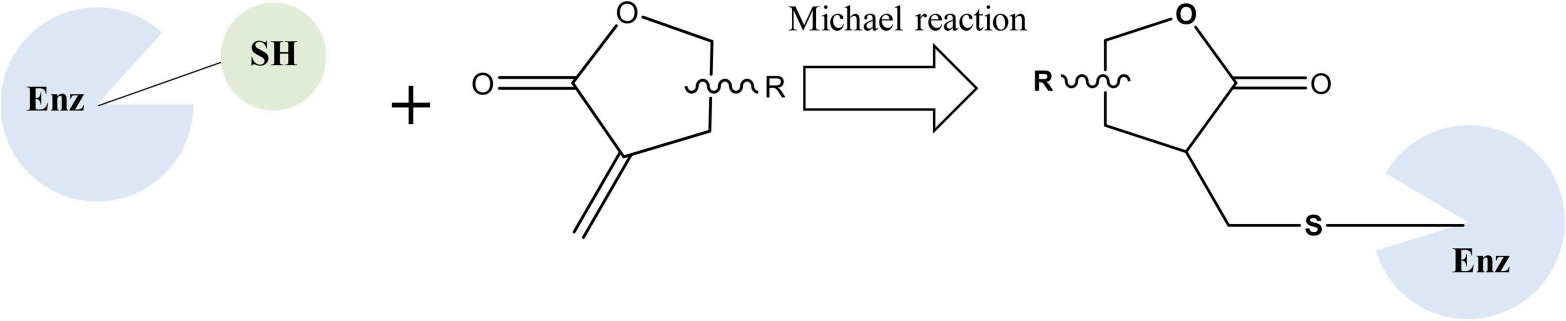
The mechanism scheme of sesquiterpene lactones.

**Figure 2:**
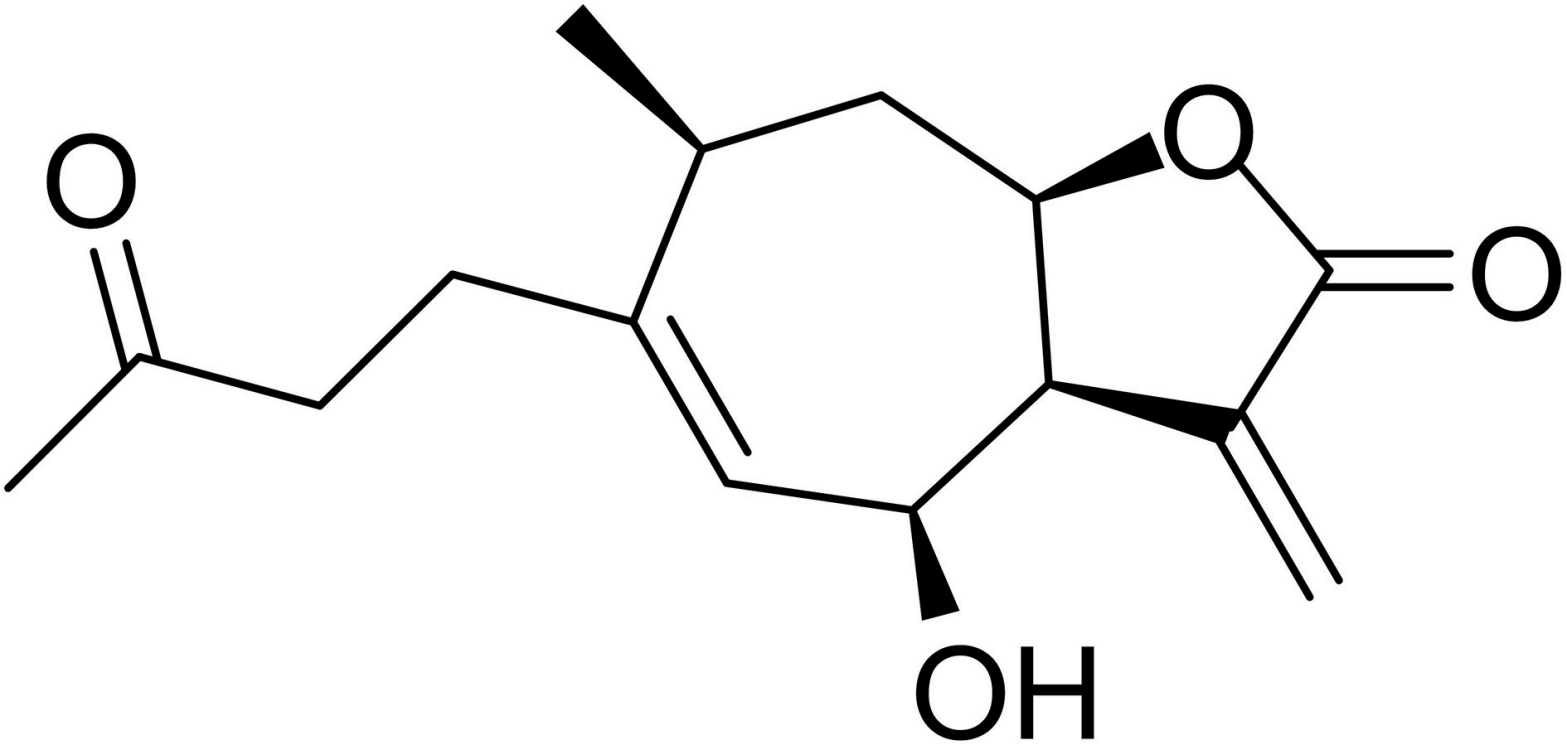
Chemical structure of HB-21.

Natural products have historically served as a major source of new leads for pharmaceutical development, especially for cancer therapy (Newman and Cragg, 2016). Sesquiterpene lactones (SLs) are one of the most prevalent secondary metabolites in plants, especially in Asteraceae (Chadwick et al., 2013). They have been subject to a number of studies because of their outstanding biological activities, particularly in antiinflammation and anticancer (Lyss et al., 1998; Whan Han et al., 2001; Gertsch et al., 2003; Chen et al., 2011). The α-methylene-γ-lactone group (αMγL) of SLs was regarded as the pharmacophore for biological effects based on its alkylation power (Chadwick et al., 2013). Much research has proven that the αMγL group is capable of covalently binding to thiol groups of available cysteines through a Michael reaction, as shown in Figure 1 (Liu et al., 2014; Wu et al., 2017). Here, we identified a natural compound 6β-Hydroxy-tomentosin (Figure 2), termed HB-21, which can bind to cysteine 473 of Cdc25B by forming a covalent bond. Furthermore, the related biological activities of cancer cells following treatment with this compound, such as the phosphorylation state of substrates and cell cycle arrest, were confirmed.

## Materials and methods

### Reagents

HB-21 (catalog no. BBP04900) was purchased from BioBioPha Co., Ltd (Kunming, China) with a purity < 95%.

### Gene expression and protein purification

The Cdc25B (372–551) coding sequence with an N-terminal TEV cleavage site was inserted into a pCold-GST vector. The protein expression was performed as described in previous research (Lund et al., 2015). In summary, the Cdc25B catalytic domain (372–551) was expressed in *E. coli* BL21 (DE3) with an N-terminal GST tag in the LB medium supplemented with 50 μg/mL ampicillin. The cells were grown at 37°C, and protein expression was induced by adding 0.5 mM IPTG until the OD_600_ reached approximately 0.6. Then, the temperature was reduced to 21°C, and the expression continued for 20 hours. The cells were collected by centrifugation at 4°C and suspended in lysis buffer (50 mM Tris, pH 8.0, 150 mM NaCl, 0.5 mM DTT, and 0.5 mM PMSF). The suspensions were lysed using ultrasonication, and the supernatant containing soluble protein was collected by centrifuging for 40 min at 19,000 rpm with a Beckman centrifuge at 4°C. The protein was captured by glutathione resin and eluted with lysis buffer containing 20–50 mM L-glutathione. The GST tag was removed by adding HRV 3C protease, and further purification was performed by S-200 size-exclusion chromatography. The purified protein was pooled and frozen at −80°C.

### *In vitro* enzymatic assay

The CycLex® Protein Phosphatase Cdc25B Fluorometric Assay Kit (CYClex, Cat. No. CY-1353) was used to screen for active compounds that inhibit the diphosphate activity of Cdc25B. The activities were measured using the substrate O-methyl fluorescein phosphate (OMFP) in a 96-well microtiter plate assay based on the manufacturer's protocol. In summary, 40 μL of assay mixture and 5 μL of test compound were combined in the wells and incubated for 15 min at room temperature with 5 μL of recombinant Cdc25B. Afterward, 25 μL of stop solution was added. Fluorescence was measured at an excitation wavelength of 485 nm and an emission wavelength of 530 nm using a fluorescence microplate reader (BioTek Instruments, Inc., Winooski, Vt, USA).

### Molecular modeling

The docking method used is described in previous work (Liu et al., 2014). In summary, molecular modeling was performed using Maestro 9.0. The X-ray structure of Cdc25B (PDB code: 1QB0) was downloaded from the Protein Data Bank (PDB, http://www.pdb.org) and prepared with “Protein Preparation Wizard” workflow using default settings. The grid-enclosing box was generated within 10 Å from the cys473 in the refined crystal structure. The structure of HB-21 was prepared using the Ligprep module. Docking was performed using the covalent docking module. The terminal carbon atom of the α-methylene moiety of HB-21 and the sulfur atom of cys473 were specified as the ligand reactive group and the receptor bond.

### Western blot

The phosphorylation status of CDK1 was analyzed by Western blotting as described in our previous work (Zhang et al., 2014). In summary, the tsFT210 cells (1 × 10^6^) were treated with HB-21 (0, 1, 5, 25 μM) for 4 h and the lysed protein was analyzed *via* 10% SDS polyacrylamide gels. The protein signals were captured with primary antibodies and secondary antibodies according to the manufacturer's instructions. In this process, the protein β-actin was used to normalize target protein. All the antibodies used in this paper were purchased from Cell Signal Technology (Inc, China). The data shown in Figure **6** are representative of two independent experiments.

### Cell cycle analysis

The method of cell cycle analysis used was referenced by others (Tsuchiya et al., 2012). Briefly, the tsFT210 cells (1 × 10^5^ cells/well) were blocked at the G2/M phase by increasing the temperature from 32 to 39°C and treating for 17 h. Then, the cells were synchronized at 32°C and immediately treated with shikonin. The cells were stained (50 μg/ml propidium iodide, 0.1% sodium citrate, and 0.2% NP-40) and analyzed by flow cytometry (BD Biosciences). The concentration of nocodazole used was 100 nM. The data shown in Figure **7** are representative of two independent experiments.

### Cell lines and culture condition

The cancer cell line tsFT210 was kindly provided by the lab of Dr. Rongcai Yue (School of Pharmacy, Second Military Medical University). The tsFT210 cells were kept at logarithmic growth in 5% CO_2_ at 37°C in the RPMI-1640 medium, supplemented with 10% FBS and 1% penicillin G-streptomycin, in a humidified chamber at 5% CO_2_.

### Mass-spectrometric analysis of HB-21 binding

The molecular weights of protein molecules and the protein–ligand adducts were detected as suggested by others (Böth et al., 2013). In brief, 5 mg of Cdc25B (372–551) was incubated with or without 1 mM HB-21 at 20°C for 30 min. Subsequently, the samples were diluted in 0.5 ml denaturing buffer [5%(v/v) acetonitrile, 0.1% (v/v) formic acid, 0.5 mM TCEP], and the molecular weights were detected by ESI-Q-TOF (Waters Corp.).

## Results

### The inhibitory effects of HB-21 on recombinant human cdc25B phosphatase

**Figure 3:**
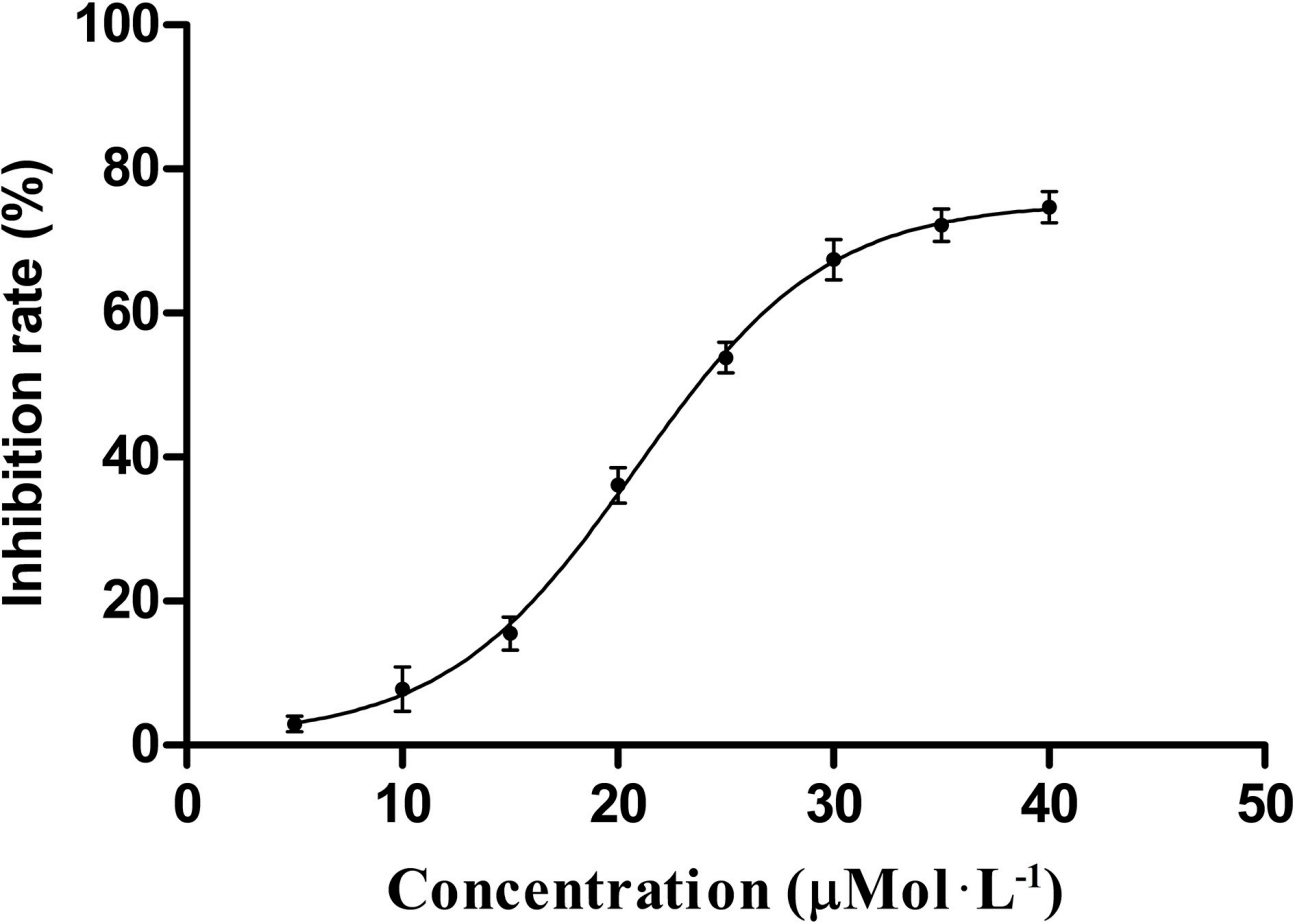
Dose–response curve for inhibition of Cdc25B by compound HB-21.

Using the protein phosphatase Cdc25 combo fluorometric assay kit, HB-21 inhibited recombinant human Cdc25B *in vitro* in a concentration-dependent manner (Figure 3) with an IC_50_ value of 24.8 ± 1.63 μM. Caulibugulone A was identified as an inhibitor of Cdc25s (Brisson et al., 2007) and thus was included as a positive control. This compound showed comparative inhibition of Cdc25B with an IC_50_ value of 5.37 ± 0.45 μM.

### Binding of HB-21 to cdc25B

**Figure 4:**
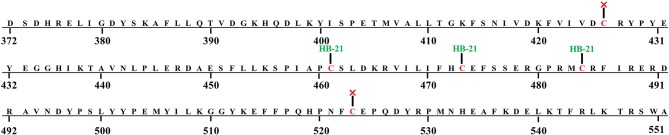
The sequence of Cdc25B (374-551) and available cysteine residues (× not accessible).

The inhibitory effect of HB-21 on Cdc25B likely occurs through covalently binding to the cysteine residues within the active site, as shown in Figure 1. Incubation of the truncated form of Cdc25B (372–551) with HB-21 led to the formation of covalent Cdc25B–HB-21 (× 3) adducts according to the results of ESI-MS (Table 1 and Supplementary Material). The 21421.76 Da peak was assigned as the molecular mass of the truncated Cdc25B (residues 372–551) because it aligned with the calculated mass (21420.52 Da) on the basis of the sequence. A new mass peak (22214.18 Da) was generated after the incubation with 1 mM HB-21, and this mass corresponds to the exact mass of the three HB-21 added to that of Cdc25B (372–551) (Table 1). There were 5 cysteines in the truncated Cdc25B (374–551) as shown in Figure 4. However, two of them (Cys426 and Cys523) were not available for the formation of covalently bonded adducts with HB-21, as they were buried under the protein surface. Therefore, three HB-21 molecules covalently bound to Cdc25B (374–551).

**Table 1:** Covalent adducts formed by Cdc25B with HB-21.

**Cdc25B(374-551) molecular mass (Da)**	**HB-21 molecular mass (Da)**	**Detected mass (Da)**	**Mass difference (Da)**
21421.76	264.1	22214.18	792.42 (3 × 264.14)

### Molecular model of HB-21 interactions with cdc25B catalytic domain

**Figure 5:**
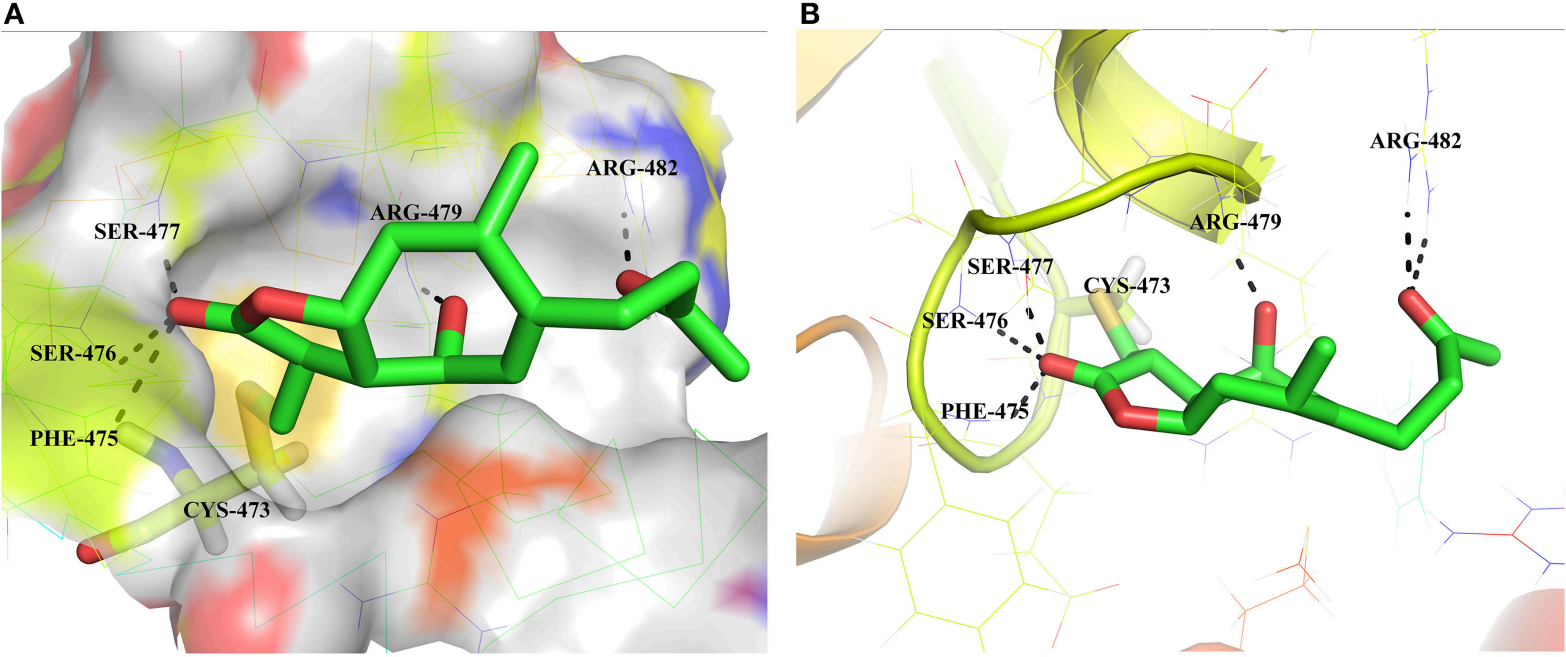
Binding mode of the HB-21 in the Cdc25B-binding cavity. **(A)** Overall view the Cdc25B is shown in surface, and the docked inhibitor is represented by green sticks. **(B)** Detailed binding interactions of compound HB-21 with Cdc25B. Key interacting residues are represented as lines. H-bonds are shown as dashed black lines. The figures were generated using Pymol (PDB ID: 1QB0).

To evaluate the binding mode and affinity of HB-21 with Cdc25B, molecular modeling was performed using the docking program Glide package. The crystal structure of Cdc25B has been solved with a resolution of 1.91 Å, showing that the catalytic domain of Cdc25B contains the canonical HCX_5_R PTPase catalytic-site motif (Reynolds et al., 1999). In this motif, C represents the catalytic cysteine 473 which forms a phosphate-binding loop with five X residues and arginine 479. In the proposed binding mode of HB-21-Cdc25B, HB-21 covalently binds to the cys473-located pocket of Cdc25B with suitable shape complementarity. In addition, six hydrogen bonds were observed between HB-21 and residues surrounding cys473 (Figure 5). Although the covalent binding between HB-21 and cys473 of Cdc25B plays a crucial role in the inhibition process, these non-covalent interactions, such as hydrogen bonds, were previously thought to increase the rate of initial site-recognition and cause a simultaneous increase in binding affinity (Liu et al., 2014).

### HB-21 inhibits CDK1 dephosphorylation and delays the entry into mitosis

**Figure 6:**
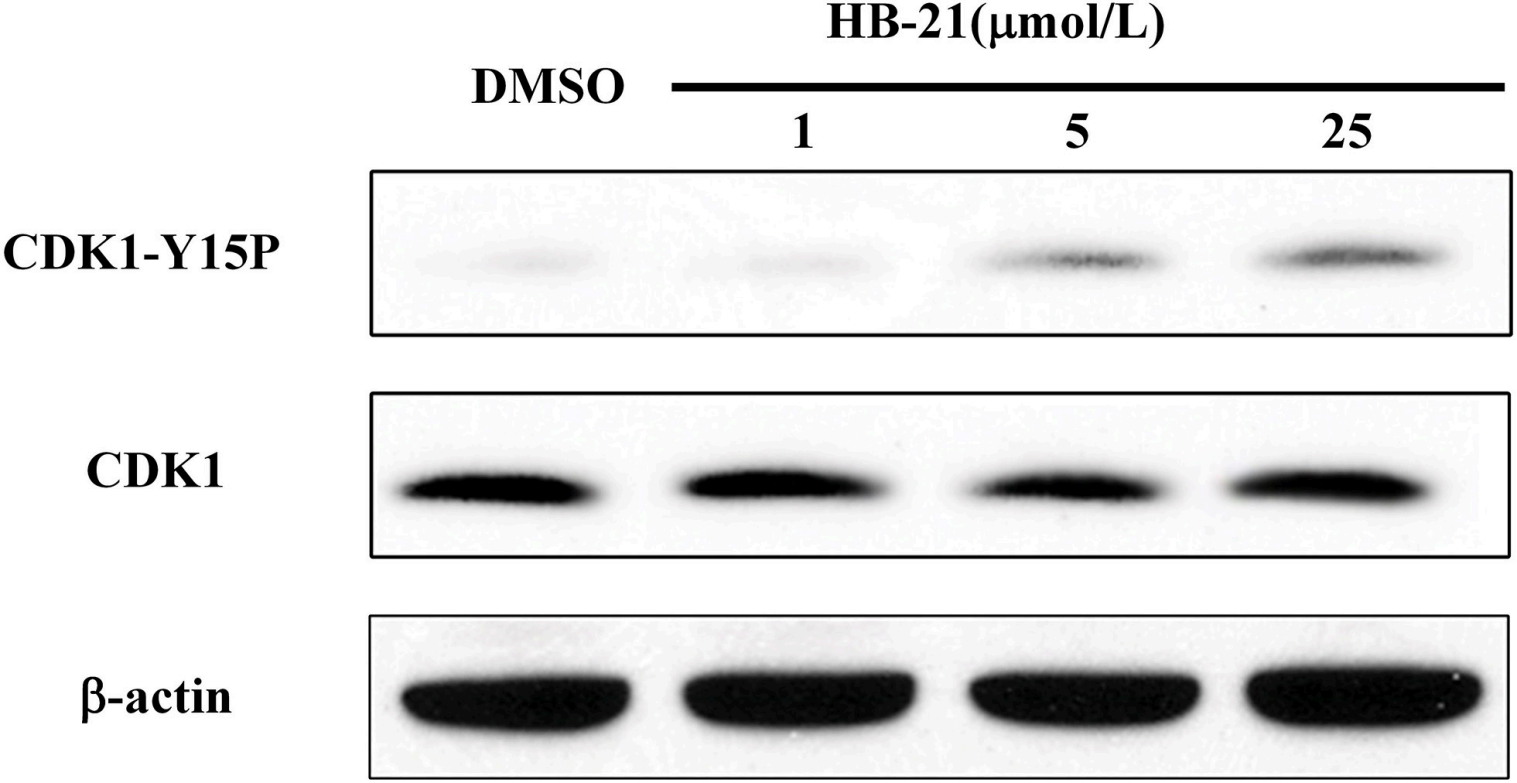
Inhibition of CDK1 dephosphorylation caused by HB-21. The cells in the G2/M phase were treated with the indicated concentration of HB-21 or DMSO for 4 h, and then harvested. The samples were processed for Western blot analysis.

Endogenous Cdc25 phosphatases control the cell cycle through dephosphorylating their natural substrate, cyclin-dependent kinases (CDKs) (Boutros et al., 2007). Therefore, the CDK1 protein will be hyperphosphorylated if CDC25s are inhibited. To confirm whether HB-21 could inhibit the activity of intracellular Cdc25 phosphatases, the phosphorylation status of CDK1 was analyzed by Western blotting. At concentrations of 5 and 25 μM, HB-21 induced an accumulation of the tyrosine 15-phosphorylated form of CDK1 (Figure 6). These results suggested that HB-21 downregulated the activity of the Cdc25B phosphatase, leading to hyperphosphorylation of CDK1 in cultured cells. Hence, the effects of this compound on cell cycle progression were examined.

### HB-21 inhibits cell cycle progression

**Figure 7:**
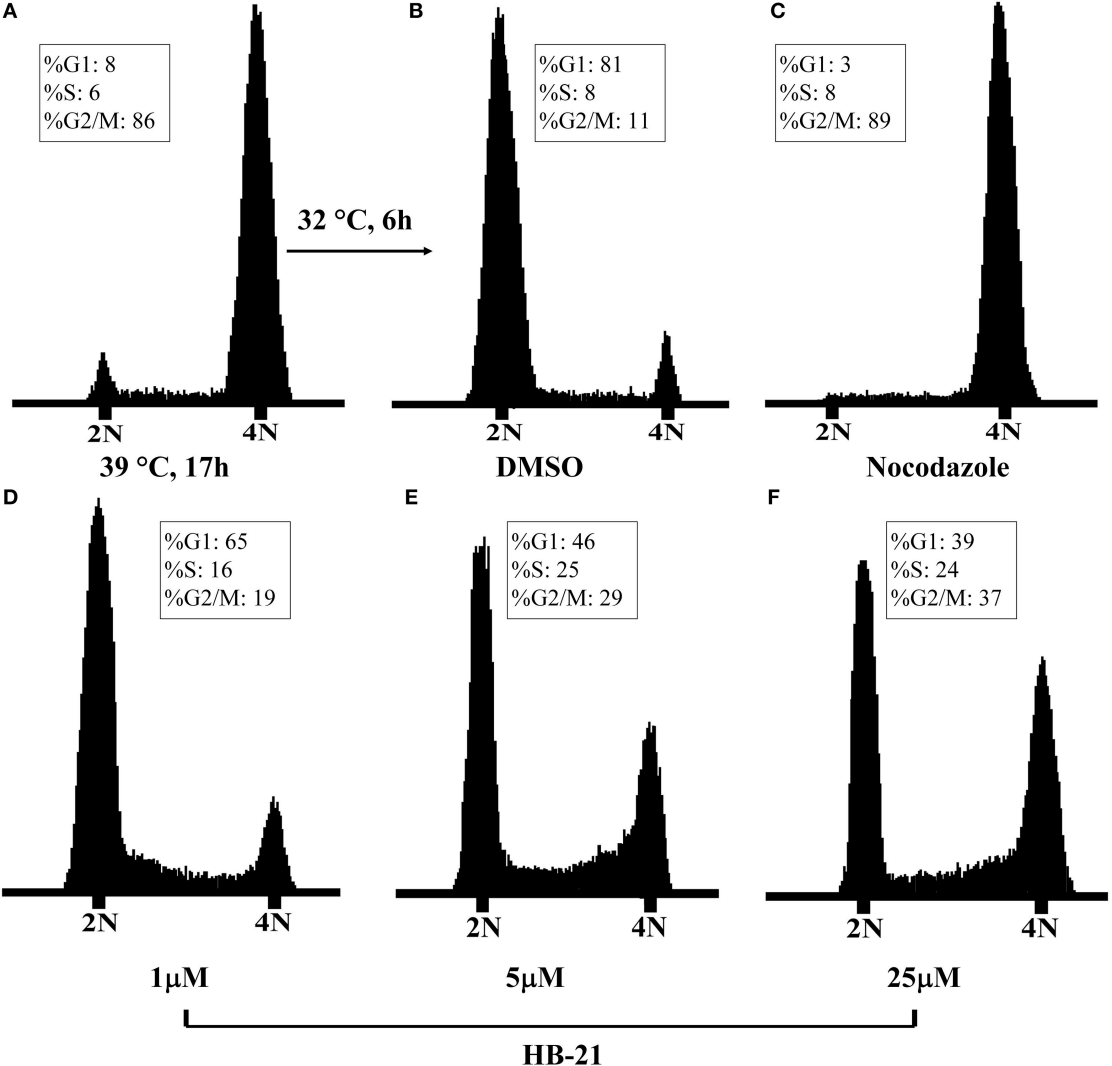
Cell-cycle analysis of tsFT210 cells in the absence or presence of HB-21. **(A)** G2/M-arrested cells after a temperature shift for 17 h at 39°C. **(B)** DMSO-treated cells after a temperature shift for 4 h at 32°C. **(C)** Cells treated with 100 nM nocodazole. **(D–F)** Cells treated with 1–25 μM HB-21.

The inhibition of Cdc25s will result in dephosphorylation of CDKs and cell cycle arrest. Therefore, the effects of HB-21 on cell cycle progression were investigated. The tsFT210 cell line has been widely used for the study of cell cycle progression because it can easily be controlled at different cell cycle phases through changing temperature (Th'ng et al., 1990). The cell cycle differences between synchronized tsFT210 cells treated with the indicated concentration of HB-21, nocodazole (a potent mitotic blocker), and 1% DMSO were analyzed by flow cytometry and shown in Figure 7. The positive control nocodazole significantly arrested the cells at G2/M phase and DMSO did not show a significant impact on cell cycle progression. Comparatively, the HB-21-treated tsFT210 cells were blocked at the G2/M phase in a concentration-dependent manner. Such results provide supplementary evidence that HB-21 can target Cdc25B and delay cell cycle progression at the Cdc25B-related G2/M phase.

## Discussion

Cys473 is the crucial cysteine for catalyzing the substrate of Cdc25B. The most crucial quinone-derived inhibitors supposedly inactivate the enzyme through oxidizing the thiolate group of cys473 (Cui et al., 2017). Until now, no inhibitors have been reported to bind directly to cys473. This study has shown that the natural product HB-21 can directly bind to cys473 by forming a covalent bond.

The structural biodiversity of natural products makes them a valuable source for drug development (Lund et al., 2015). Studies on SLs have increased due to their prevalence in plants and their diverse bioactivity (Chadwick et al., 2013). HB-21 belongs to a type of xanthane sesquiterpene, which possesses an αMγL moiety. The multiple bioactivities of SLs are attributed to the αMγL unit, which has the potential to bind to thiol groups of proteins covalently by a Michael reaction (García-Piñeres et al., 2001). Using mass spectrometry and molecular modeling, this investigation has also proven that this mechanism exists between HB-21 and Cdc25B. Modeling results showed that, in addition to the αMγL unit, other chemical groups of HB-21 are likely to have an influence on the activity of Cdc25B through non-covalent interactions. These interactions might serve as an initial site-recognition step during the binding of HB-21 to Cdc25B. The shape and size of the binding pocket of target proteins is variable, and good shape complementarity between SLs and target proteins is crucial for activity. Therefore, SLs containing more flexible groups show increased activity (Chadwick et al., 2013). Moreover, the residues around cysteine were thought to play a role in the initial site-recognition for ligand binding through other intermolecular forces, such as Van der Waals forces, hydrogen bonds, etc. (Liu et al., 2014). This outcome also explains why HB-21 showed a moderate inhibitory activity for Cdc25B. Although HB-21 covalently binds to cysteine 473, the noncovalent interactions between Cdc25B and HB-21 may be weak, resulting in slow initial site-recognition. Further research is necessary to fully understand the activity of HB-21 in the HB-21: Cdc25B co-crystal structure at the molecular level. This would allow a new HB-21-based derivative with much higher biochemical activity to be designed.

Further research also needs to address the selectivity of HB-21 for Cdc25B, and whether there are additional molecular targets for HB-21 within the cell. In this study, HB-21 began to induce cell cycle arrest at a concentration of 5 μM in G2/M phase cells. At this concentration, however, HB-21 has a relatively low inhibitory effect (< 5% inhibition). Three possible reasons may explain this result. (1) Only the reduced state of the cysteine's sulfhydryl group (-SH) can covalently bind to HB-21. This sulfhydryl group is, however, easily oxidized in air, resulting in the inability of HB-21 to bind to Cdc25B. (2) The Michael reaction needs time to complete. The Cdc25B and HB-21 were, however, only incubated for 15 min to keep the protein in a reduced state. However, in addition to the aforementioned reasons, the low inhibitory effect also suggests that HB-21 has other intracellular targets, such as the other homologues of Cdc25s (Cdc25B, and -C) sharing common structural properties with Cdc25A, especially for the signature motif (HCxxxxxR), which will be necessary to confirm in future research.

In conclusion, this study has identified a new type of Cdc25B inhibitor, HB-21. HB-21 resulted in the dephosphorylation of Cdc25's natural substrate, CDK1, and the inhibition of cell cycle progression by HB-21 covalently binding to cys473, located within the active site of Cdc25B. Neither *in vivo* nor *in vitro* activity of HB-21 has been evaluated prior to this study. This is the first time that HB-21 has been found to have anticancer activity, allowing for HB-21 to provide a new molecular template for anticancer drug development. The *in vivo* studies will be part of our future studies.

## Author contributions

SZ, QG, and ZS designed the experiments. QJ, QG, XF, and YW performed the experiments and analyzed data. SZ wrote the manuscript. ZS edited the manuscript. All the authors read and approved the final manuscript.

### Conflict of interest statement

The authors declare that the research was conducted in the absence of any commercial or financial relationships that could be construed as a potential conflict of interest.
