# Corrigendum: Dual-specificity phosphatase CDC25B was inhibited by natural product HB-21 through covalently binding to the active site

**DOI:** 10.3389/fchem.2022.1082236

**Published:** 2022-11-18

**Authors:** Shoude Zhang, Qiangqiang Jia, Qiang Gao, Xueru Fan, Yuxin Weng, Zhanhai Su

**Affiliations:** ^1^ State Key Laboratory of Plateau Ecology and Agriculture, Qinghai University, Xining, China; ^2^ Department of Pharmacy, Medical College of Qinghai University, Xining, China

**Keywords:** Cdc25B inhibitor, sesquiterpene lactone, anticancer, cell cycle progression, covalent binding to protein

In the original article, there was an error in the affiliations as published, in which a third affiliation was wrongly included for author “Shoude Zhang”. The correct affiliations appear above.

In the original article, there was an error in Figure 6, page 4, as published. In the same batch of experiments, WB experiments for two compounds were done at the same time, and the article incorrectly used the results of another drug in the experiments. The corrected Figure 6 and its caption appear below.

**FIGURE 6:**
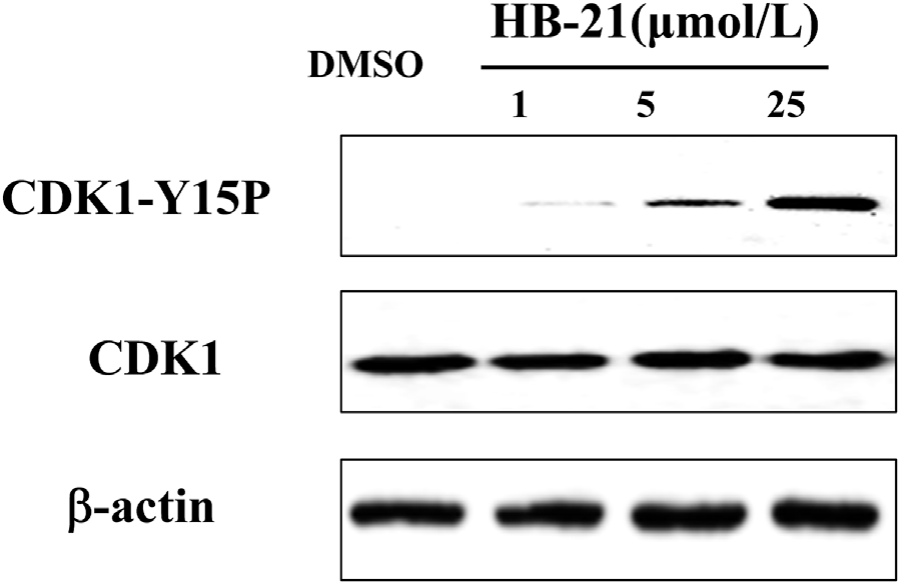
Inhibition of CDK1 dephosphorylation caused by HB-21. The cells in the G2/M phase were treated with the indicated concentration of HB-21 or DMSO for 4 h, and then harvested. The samples were processed for Western blot analysis.

The authors apologize for these errors and state that this does not change the scientific conclusions of the article in any way. The original article has been updated.

