# Supplementary material for: Dual-Specificity Phosphatase CDC25B Was Inhibited by Natural Product HB-21 Through Covalently Binding to the Active Site
Source: Front Chem. 2018 Nov 13;6:531. doi: 10.3389/fchem.2018.00531 (PMC6282036; doi:10.3389/fchem.2018.00531)

Figure S1: Mass spectrometric analysis of the Cdc25B segment (residues 374-551) with and without HB-21 (264.1 Da). The mass difference in the major peaks corresponds very closely to the mass of three HB-21 (792.3 Da).


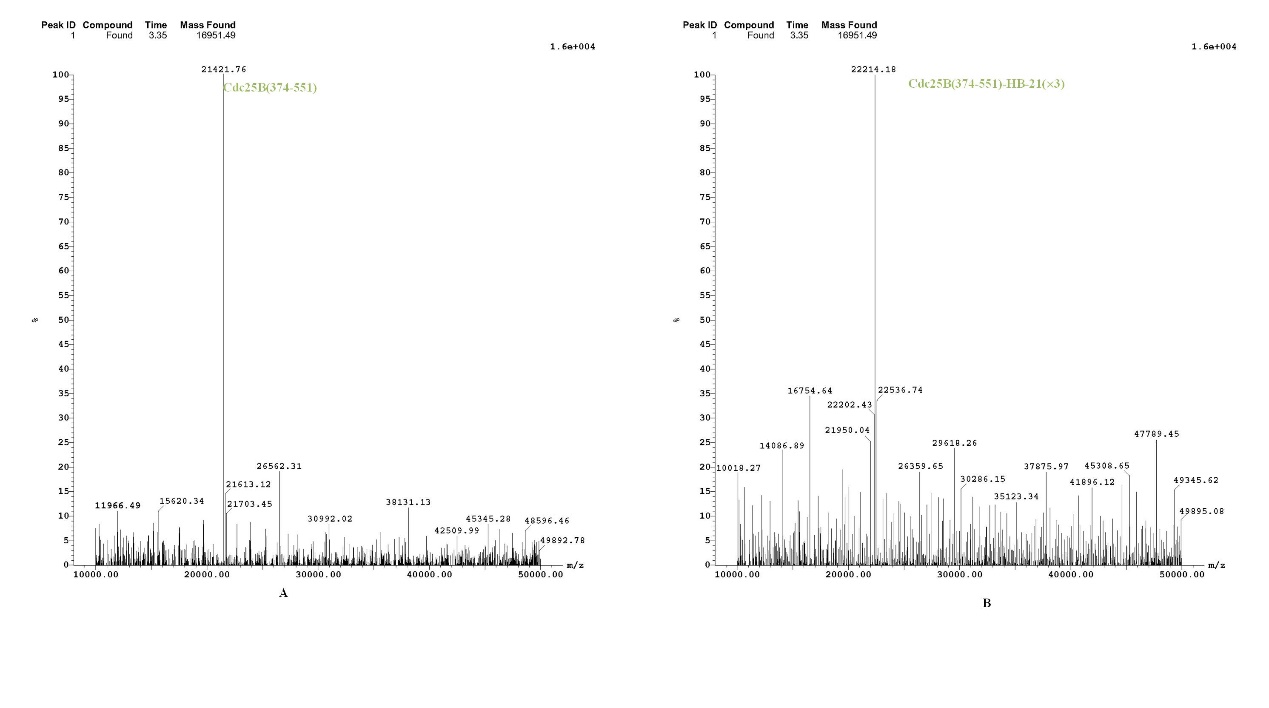

Supplement: Supplementary file 1 [file Data_Sheet_1.docx]
